# Improved electrochemical performance of bio-derived plasticized starch/ reduced graphene oxide/ molybdenum disulfide ternary nanocomposite for flexible energy storage applications

**DOI:** 10.1038/s41598-023-48326-8

**Published:** 2023-11-28

**Authors:** Eashika Mahmud, Muhammad Rakibul Islam

**Affiliations:** Department of Physics, Bangladesh University of Engineering and Technology (BUET), Dhaka, Bangladesh

**Keywords:** Energy storage, Bioinspired materials, Two-dimensional materials

## Abstract

A ternary nanocomposite of plasticized starch (PS), reduced graphene oxide (rGO), and molybdenum disulfide (MoS_2_) was prepared via a solution casting process, with MoS_2_ concentrations ranging from 0.25 to 1.00 wt%. The structural, surface morphological, optical, and electrochemical properties of the nanocomposites were studied. FTIR analysis reveals the formation of new chemical bonds between PS, rGO, and MoS_2_, indicating strong interactions among them. The XRD analysis showed a reduction in the crystallinity of the nanocomposite from 40 to 21% due to the incorporation of nanofiller. FESEM micrograph showed an increment of the surface roughness due to the incorporation of rGO-MoS_2_ layers. UV–vis spectroscopy demonstrated a reduction of optical bandgap from 4.71 to 2.90 eV, resulting from enhanced charge transfer between the layers and defect states due to the addition of nanofillers. The incorporation of MoS_2_ increase the specific capacitance of the PS from 2.78 to 124.98 F g^−1^ at a current density of 0.10 mA g^−1^. The EIS analysis revealed that the nanofiller significantly reduces the charge transfer resistance from 4574 to 0 Ω, facilitating the ion transportation between the layers. The PS/rGO/MoS_2_ nanocomposite also exhibited excellent stability, retaining about 85% of its capacitance up to 10,000 charging-discharging cycles. These biocompatible polymer-based nanocomposites with improved electrochemical performance synthesized from an easy and economical route may offer a promising direction to fabricate a nature-friendly electrode material for energy storage applications.

## Introduction

The world's energy consumption is soaring due to a growing population, economic expansion, and technological advancements across industries, transport, and urban development. However, this surge has led to increased use of finite fossil fuels, emitting hazardous gasses and contributing to global warming and environmental issues^1^. As a cleaner alternative, renewable energy sources have gained attraction^2^. Yet, their intermittent nature makes them unpredictable. Energy storage solutions have emerged as vital tools for stabilizing supply and demand by capturing excess electricity at the peak production time and storing it for later use, ensuring a stable and secure electricity supply. Over the past few decades, a multitude of energy storage devices, such as lithium batteries, supercapacitors, and alkali-metal ion batteries, have been developed to effectively store and utilize these energies as required. To conserve natural integrity, the constituent materials of energy storage devices must be biocompatible and biodegradable. Various materials, including 2D materials^3^, nanotubes^4^, metal oxides^5^, transition metals^6^, polymers^7^, and biopolymers^8^, have been effectively employed for storing energy due to their diverse microstructural properties. Apart from nature conservation, however, many of these materials are expensive and require complex manufacturing processes. While starch is not only eco-friendly, it also has ample resources and is easily extractable from various plant sources, including potato, corn, cassava, and even some microorganisms^9^. The source materials for the starch can be obtained at a very cheap rate of less than $1/kg worldwide. The source materials' easy availability and simple extraction process, make starch a cost-effective choice for the biopolymer matrix.

However, starch films generally exhibit poor charge conductivity, low solubility in various electrolytes, low mechanical properties, and high-water affinity, which limit their commercial use in electronics^10^. To address these shortcomings, various approaches have been attempted, including chemical modification, blending with cellulose or starch crystals, agar, maltodextrin, xanthan, arabinoxylan, chitosan, and the addition of plasticizers (such as glycerol, sorbitol, and lignin)^11–13^. Some research has focused on the development of composites that incorporate fillers, such as starch/ZnO^14^, PAni-starch^15^, starch-rGO^16,17^, and others^18^, to enhance the specific properties. The incorporation of rGO into starch was found to improve the capacitive performance of starch, but the addition of more rGO reduces the electrochemical performance due to the agglomeration of rGO layers^17^. This hinders the possibility of obtaining improved electrochemical performances together with higher specific capacitances from the rGO-incorporated PS matrix. Therefore, one possible route to obtain improve the electrochemical performance of PS/rGO nanocomposite could be the incorporation of a second nanofiller into it by keeping the concentration of the rGO nanofiller low. To the best of our knowledge, no such attempts were made to enhance the electrochemical performance of PS/rGO nanocomposite by incorporating a second nanofiller. The unique 2D structure of transition metal dichalcogenide (TMDs) is a suitable candidate as the second nanofiller for its complementary layer structure and potential synergies. More so, TMDs exhibit tunable band gaps, valley polarization, and strong light-matter interactions and can enhance catalytic activity, charge transport properties, and more^19–21^.

Among the different choices of TMDs nanofillers, nanostructured MoS_2_ has attracted significant attention among the filler materials of nanocomposites due to its excellent properties, including a narrow band gap (1.75 eV), visible light absorption, large surface area, and unique stacked morphology with strong covalent bonds^22,23^. Incorporation of MoS_2_ with rGO may facilitate charge transport through their inter- and intra-layer interactions, leading to faster ion diffusion between the layers and electrolytes^24^. Consequently, the hybrid layer structure can produce high specific capacitance, high energy density, and improved charge-storage capacity^25^. Using MoS_2_ alone may provoke the shuttle effect due to the dissolution of polysulfides in the energy storage performance^26^. Combining rGO and MoS_2_ with immobilized support through PS can resolve the dissolution of polysulfides^27,28^. Therefore, there is a high demand of developing polymer-based ternary nanocomposites by incorporating both MoS_2_ and graphene nanofillers. To achieve this, a synthesis method that uses mild reaction conditions is time-efficient and requires low energy consumption, which is highly desirable.

In this work, a simple and economical approach was used to enhance the electrochemical performance of the biodegradable PS matrix. A novel ternary PS/rGO/MoS_2_ nanocomposite has been synthesized via a facile solution casting technique. The solution casting method provides a convenient and cost-effective approach for producing large-scale polymer-based nanocomposites suitable for industrial applications^29^. The distinct characteristics of the nanocomposite films were probed using Fourier-transform infrared (FTIR) spectroscopy, X-ray diffraction (XRD), field emission scanning electron microscopy (FESEM), contact angle (CA) measurements, and analysis of optical properties. The capacitive attributes were assessed through cyclic voltammetry and galvanostatic charging-discharging, coupled with electrochemical impedance spectroscopy (EIS) analysis utilizing an equivalent AC circuit. Enhanced specific capacitance (increased from 2.78 to 124.98 F g^−1^ at 0.10 mA g^−1^), remarkable capacitive retention (maintaining 85% capacity after 10,000 cycles), and reduced contact resistance (0 Ω) were observed in the electrochemical assessment. This environmentally friendly electrode material, sourced from nature, non-hazardous, and biodegradable, exhibits improved energy storage properties. The complete study of this biomaterial with improved electrochemical properties may be used in the field of flexible bio-friendly next-generation electronic devices.

## Materials and characterization

All methods were carried out in accordance with relevant guidelines and regulations.

### Materials

Natural flake graphite (98%, Loba Chemie, India), potassium permanganate (KMnO_4_) (99%, Merck, Germany), sodium nitrate (NaNO_3_) (99%, Qualikems, India), concentrated sulfuric acid (H_2_SO_4_) (98%, Merck, Germany), hydrogen peroxide (H_2_O_2_) (30%, Merck, India), glycerol (Propane-1, 2, 3-triol) (Merck, India), sodium molybdate dehydrates (Na_2_MoO_2_·2H_2_O) (98%, Merck, Germany), thiourea (CH_4_N_2_O) (98%, Research-Lab, India), dimethyl sulfoxide (DMSO, C_2_H_6_OS) (99.5%, Merck Germany).

### Sample preparation

#### Extraction of starch

The fresh potato purchased from the local market was cleaned, peeled, and grated. The grated potato was mixed with DI water in a beaker to remove debris. Stirring separated the starch particles, which settled as sediment. The crumpled potato was removed before the liquid changed color. After 15 min, the white milky starch settled in the beaker. The starch was collected, washed, dried at 60 °C, and ground to obtain a fine powder. Please see the Supplementary section for the details.

#### Synthesis of reduced graphene oxide

Graphene oxide (GO) was prepared before reducing oxygen from it to obtain reduced graphene oxide (rGO). GO was prepared by the modified Hummer method, which involves the oxidation and exfoliation of graphite^30^. Then rGO was synthesized by using the hydrothermal method. In this method 5 mg of GO powder was added to 15 mL of DI water to form a 0.3 mg/mL solution, which was sonicated for 30 min. The dispersion was transferred to a Teflon-lined stainless-steel autoclave, heated at 150 °C for 6 h, and cooled naturally. The product was washed, dried at 40 °C overnight, and ground to obtain homogeneous rGO powder.

The XRD spectrum of rGO is presented in Supplementary Fig. S2a. The well-resolved broad diffraction peaks of the (0 0 2) and (0 0 1) planes were observed at 2θ of 24.8°, and 43.12°, indicating the formation of rGO. The broad peak at (0 0 2) corresponds to a few layers of rGO sheets in the particles. The peak at 2θ of 43.12° in rGO is related to the intercalation of oxygen groups or water molecules, causing an increase of interlayer spacing.

For the rGO spectrum, the intense peak located at 1542 cm^−1^, 1689 cm^−1^ can be attributed to the stretching bands C=C, C=O, respectively. The very weak peak located at 2321 cm^−1^ corresponds to the C–O stretching. In addition, the broad peak at 3433 cm^−1^ indicates the presence of –O–H bond^31^.

#### Synthesis of molybdenum disulfide

Molybdenum disulfide (MoS_2_) was also synthesized by hydrothermal method. First, Sodium molybdate dihydrate (Na_2_MoO_2_·2H_2_O) and thiourea (CH_4_N_2_O) were dissolved in 120 mL distilled water. The solution was stirred vigorously until the solution became transparent, stirring increases the reaction rate. The transparent solution was transferred to an airtight Teflon jar for high-pressure treatment in an autoclave at 200 °C for 24 h, yielding a blackish precipitate. After centrifugation with DI water and ethanol, the precipitate was dried at 60 °C and ground to obtain fine MoS_2_ powder.

The XRD spectrum of MoS_2_ is presented in Supplementary Fig. S3a. In the XRD spectrum, the diffraction peaks at 2θ values of 13.99, 33.14, 39.46, 40.8 and 58.77 are observed that correspond to the (0 0 2), (1 0 0), (1 0 3), (1 0 5) and (1 1 0) reflections of the hexagonal structure of MoS_2_ respectively^32^.

The FTIR spectrum of MoS_2_ is presented in Supplementary Fig. S3b. The peak at 620 cm^−1^ can be attributed to the Mo–S stretching vibration. The multiple peaks at 700–1150 cm^−1^ can be assigned to the sulfate groups, while the sharp peaks at 3440 and 1610 cm^−1^ can be ascribed to the O–H stretching and water bending, respectively^33^.

#### Preparation of nanocomposite

To prepare the PS/rGO/MoS_2_ nanocomposite, at first, an aqueous solution of rGO/MoS_2_ was made, followed by sonication with DMSO as a 1:3 ratio of nanofiller and DMSO to have a uniformly dispersed solution. Three different concentrations of nanofillers were used, where rGO concentration remained fixed (1%), and MoS_2_ concentration varied from 0.25 to 1%. To prepare the film, sonicated rGO/MoS_2_, 5 g starch powder, and DI water were mixed. Then, this blend was stirred. Glycerol was added into the fine mixed solution and stirred at 90 °C. When the solution was dense enough to form a film, it was poured into a petri dish for drying at 50 °C. As biofilm is easily attracted by fungus and humidity, the dried biofilm was preserved in a warm environment. Finally, five different films had been prepared with varying concentrations of MoS_2_. Throughout this paper, nanocomposites A, B, C, D, and E denote the Plasticized Starch (PS), PS/rGO (1%), PS/rGO/MoS_2_ (0.25%), PS/rGO/MoS_2_ (0.5%) and PS/rGO/MoS_2_ (1%) nanocomposite respectively.

### Characterizations

After their synthesis, various characterization methods were used to analyze the physical properties of the PS/rGO/MoS_2_ nanocomposites. Fourier Transform Infrared Spectroscopy (FTIR) spectra of the as-prepared nanocomposite were studied in the range of 400–4000 cm^−1^ spectra to investigate the presence of the functional group. The FTIR analysis was conducted using a Shimadzu IRSpirit spectrophotometer in attenuated total reflection (ATR) mode. X-ray diffraction (XRD) analysis has been done to check the crystalline phase by using 3040XPert PRO, Philip system with monochromatic CuK_α_ (λ = 1.54 Å) radiation at a scanning rate of 2°/min from 5° to 90°. The microstructure and surface morphology were analyzed by the Field Emission Scanning Electronic Microscope using JSM-7600F, JEOL FESEM machine at an accelerating voltage of 5 kV. The optical properties were studied using a SHIMADZU UV 2600 spectrometer, where BaSO_4_ was used for baseline correction in the wavelength range of 200–900 nm at ambient conditions. The contact angle was assessed with a contact angle meter (Apex, India) through the sessile drop technique under room temperature and controlled humidity conditions. A 10 µL water droplet was employed for the measurements on the film surface. Three separate measurements of the contact angle were taken on distinct locations of the surface for each film, and the resulting average value was adopted. The electrochemical characteristics were assessed using a CS310 Electrochemical Working Station (corrtest, China) in a three-electrode configuration. This setup comprised a glassy carbon electrode employed as the working electrode, a counter electrode consisting of a platinum foil plate (1 cm × 1 cm), and a reference electrode of Ag/AgCl. Additionally, a 0.1 M KCl solution was used as the ionic aqueous electrolyte.

To prepare the homogenous slur of electrode material, first, the surface area of the working electrode was wiped with ethanol. This exposed area is where the material will be deposited and dried at 60 °C for around 1 h. In the meantime, a tiny portion of the film (a few mm^2^) was cut, then measured, and took only the desired mass. After that, the selected portion of the film was heated and stirred with DI water until the film melted and became sticky. This slurry composite was then deposited on the electrode with a micropipette. This deposited electrode was dried at room temperature for a few hours to gluttonize on the surface.

## Results and discussion

### Chemical bonding properties

**Figure 1:**
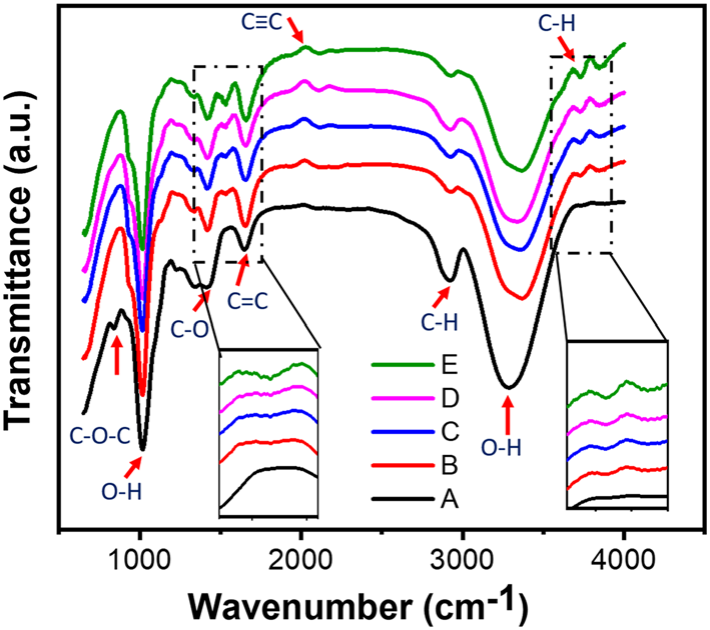
FTIR spectra of PS/RGO/MoS_2_ nanocomposite with the variation of MoS_2_ filler content. Inset represents the zoom in image for the selected portion of the FTIR spectrum.

FTIR study was performed to assess the chemical changes in PS due to the addition of rGO and MoS_2_ nanofiller. Figure 1 illustrates the FTIR spectra of all the nanocomposite ranges from 500 to 4000 cm^−1^. For curve A (PS), the peak at 837 cm^−1^ 1026 cm^−1^ corresponds to the ring vibration and asymmetric stretching C–O–C, respectively, and are a signature of the organic starch polymer^34^. The broad peaks at 1026 cm^−1^, and 3279 cm^−1^ correspond to the O–H stretching and bending. The absorption bands observed in the 1382 cm^−1^ and 1647 cm^−1^ revealed the presence of carboxylic and epoxy groups, specifically C–O bending and C=O stretching. The peak at 2929 cm^−1^ is attributed to the C–H stretching. For the PS/rGO, the C–O–C peak at 837 cm^−1^ diminished. This is attributed to the fact that in the vicinity of the starch granule, parts of its carbon ring bonds were removed, and oxygen groups were substituted by hydroxyl groups^13,35^. In the FTIR spectra, three new peaks at 1522 cm^−1^, 2017 cm^−1^, and 3682 cm^−1^ have been found due to the addition of rGO, which corresponds to C–H bending, C=C bending, and C–H stretching, respectively.

For curve E (PS/rGO/MoS_2_), the peak intensity at 1522 cm^−1^, and 2017 cm^−1^ gradually increased with the increase of MoS_2_ concentration. This suggests that improved interaction between the nanofiller and the PS matrix occurred due to the addition of nanofillers. Due to the addition, the intensity of the O–H bond gets reduced and the peak was found to be shifted towards a higher wavenumber. The intensity reduction and this shifting of peak come from the relative contribution of overlapped bands and the change of surface chemistry with the change of internal orientation due to the addition of nanofillers.

The presence of different polar groups (–COOH, C=O) and the presence of water (–OH functional group) suggests the strong intercalation between the electrolyte and the nanocomposite, which is one reason for the improved charge storing performance.

### Structural properties

**Figure 2:**
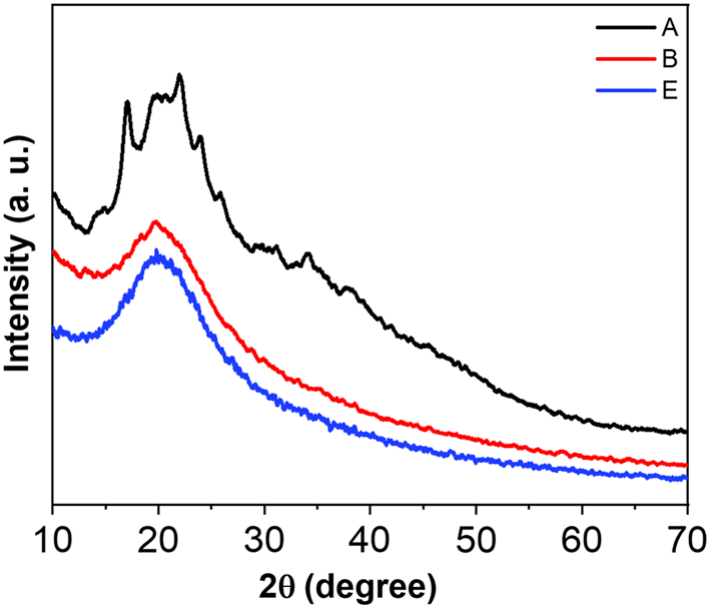
X-ray diffraction (XRD) spectra of PS, PS/rGO and PS/rGO/MoS_2_ nanocomposite.

The microstructure of the nanocomposites is investigated by analyzing their XRD spectra, which are presented in Fig. 2. The characterization peak at 2θ values of 14.86°, 17.09°, 19.88°, 22.04°, 24.01°, 25.89°, 31.19°, 34.12°, and 37.89° confirms the B-type crystalline structure of starch^36^. In the XRD spectra of PS/rGO, the tiny peaks of starch disappeared, which indicates the bonding between PS and rGO occurs. With the incorporation of MoS_2_, the intensity of the peaks gets reduced further. This suggests a reduction in the crystallinity of the nanocomposite due to the addition of nanofiller. To evaluate the effect of nanofiller on the crystallinity of the polymer matrix, the degree of crystallinity of the samples was measured using the Hermans–Weidinger approach^37^:

1 $$X_{c} = \frac{{A_{c} }}{{A_{c} + A_{a} }} \times 100$$

where *A*_*c*_ = Crystalline peak area, *A*_*a*_ = Total area.

The degree of crystallinity obtained for the different nanocomposites is presented in Table 1, which clearly shows a reduction in crystallinity due to the introduction of rGO and MoS_2_ nanofiller. The incorporation of rGO reduces the crystallinity of PS from 40 to 29%. These reductions are due to the gelatinating of PS/rGO nanocomposites and the swelling of rGO into the starch chain through the interaction of hydrogen bonds. This swelling reordered the molecular arrangement of the polymer and together with the demobilization capability of hydrogen bond, reduced the crystallinity^38^. The sheet-like structure of rGO hinders the molecular movement of PS during crystallization, which is also responsible for the decrease in crystallinity.

**Table 1:** Area under the main peaks, crystallinity, d-spacing, full width half maxima (FWHM), average crystallite size, and micro-strain of the as-prepared composites.

Sample	Area of main peaks	Crystallinity (%)	d-spacing (nm)	Full width half maximum (β)	Microstrain (*ε*)	Crystallite size (L) (nm)
PS	636	40	4.03	0.67	15.03	12.63
PS/rGO (1%)	531	29	4.45	0.60	14.95	14.05
PS/rGO/MoS_2_ (1%)	347	21	4.45	0.36	8.97	23.42

Adding MoS_2_ further reduces the crystallinity of the nanocomposites (from 29 to 21%). When crystalline materials are added to the amorphous chain molecule, the small crystalline regions of ordered structure within an amorphous matrix are destroyed. The hexagonal MoS_2_ can introduce sulfur vacancy, and the stacked layer creates dislocations in the starch matrix. These defects and dislocation can act as sites of stress concentration and hinder the growth of crystalline regions. As a result, the overall crystallinity of the starch decreases.

The d-spacing, crystallite size, and the micro-strain of the nanocomposites were also estimated from the XRD data and are tabulated in Table 1. The d-spacing can be estimated by Braggs law^39^:

2 $$n\lambda = 2dsin\theta$$

3 $$d = \frac{n\lambda }{{2sin\theta }}$$

Crystallite size and Lattice strain are also studied using the Debye–Scherrer approach^40^. Crystallite size refers to the size of the individual crystalline domains or grains within a polycrystalline material. The crystallite size was also studied following the relation:

4 $$L = \frac{0.94\lambda }{{\beta cos\theta }}$$

where $\beta$ is the full-width half maximum (FWHM).

Crystallite size provides information about the presence of imperfections, defects, or boundaries between different grain domains^41^. The crystallite size for PS is 12 nm, which increases up to 23 nm due to the incorporation of MoS^2^ in the polymer matrix. Larger crystallites can result in fewer grain boundaries, reducing the impedance to the flow of electrons, and thus lowering electrical resistance, which helps improve the charge carrier transportation^42^.

The lattice strain of the nanocomposites was calculated by the following relation:

5 $$\varepsilon = \frac{\beta }{4tan\theta }$$

Lattice strain is the broadening of the XRD peak of crystal structure due to imperfection and distortion.

### Surface morphology analysis

**Figure 3:**
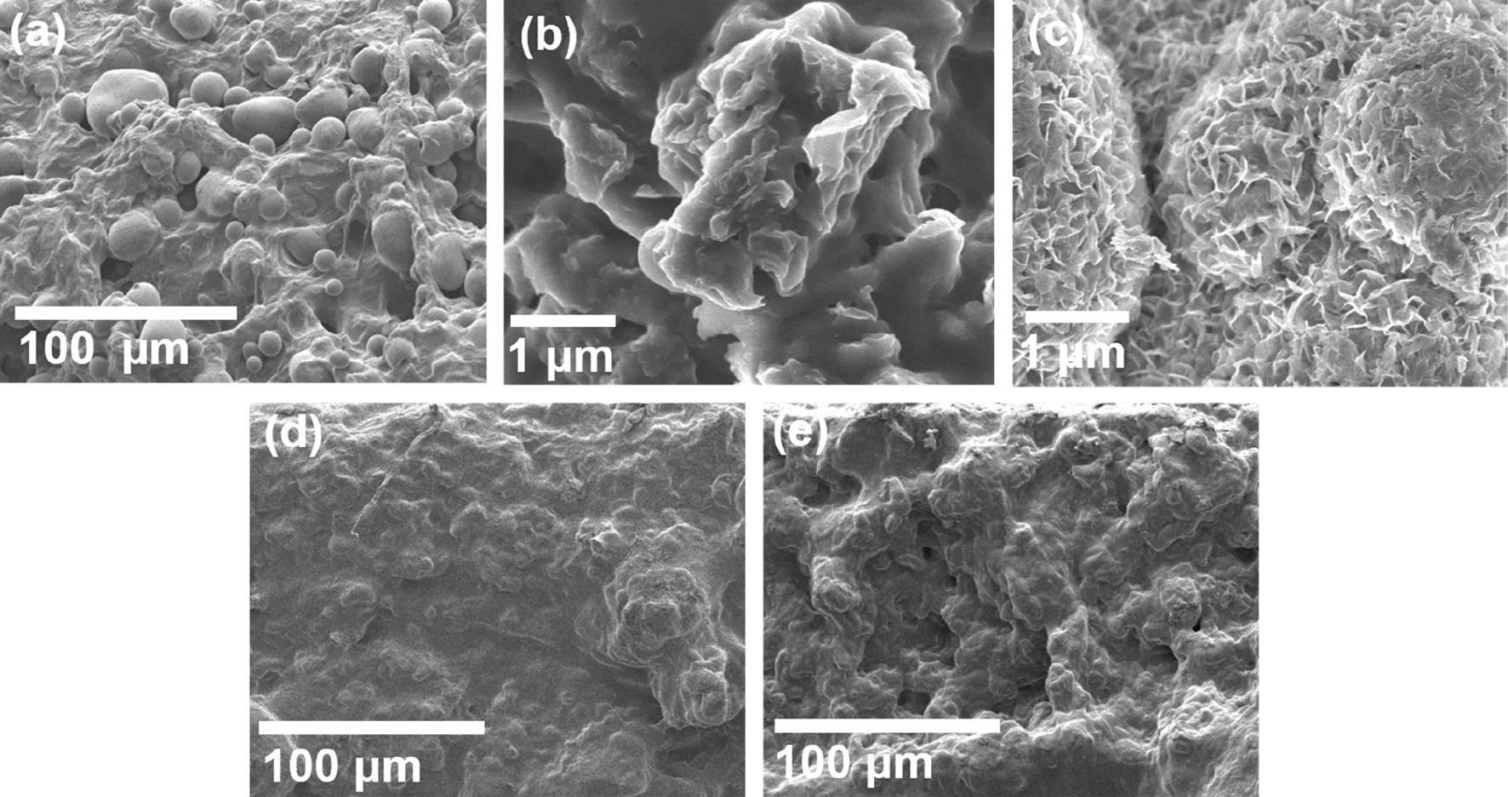
FESEM images of (**a**) PS (**b**) rGO (**c**) MoS_2_ nanoflower (**d**) PS/rGO, and (**e**) PS/rGO/MoS_2_ nanocomposite.

The FESEM study was performed to understand the morphology of the PS/rGO/MoS_2_ nanocomposites, which is presented in Fig. 3. The FESEM image of PS (in Fig. 3a) shows that it consists of different shape of granules, which confirm the B-type of the starch^43^. Figure 3b represented the FESEM micrograph of rGO consisting of a thin sheets-like structure. The rGO sheets are randomly aggregated containing wrinkled textures with distinct folding edges. Figure 3c shows the FESEM images of MoS_2_ microspheres composed of numerous thin petals that aligned themselves to a common center to form the shape of a flower. The petals of the nanoflower are several nm thick, and the diameter of flowers varies between 2 and 5 μm^44^.

The FESEM image of the cross-section of the PS/rGO is presented in Fig. 3d. The granules structure of PS disappears after the addition of rGO and is reformed into a thin sheet-like structure which is randomly aggregated. This emphasized the uniform dispersion of rGO in the PS matrix. The deformation comes from the contribution of rGO nanosheets as distinct edges, wrinkled, and folded surfaces, and the SP^2^ hybridization is present within. The folded structure of PS/rGO is also the result of the loss of the oxygen functional group, which comes from the exfoliation process of GO. In Fig. 3e, for the PS/rGO/MoS_2_, a more folded layer network is observed, which suggests a more compact nature of the composite followed by self-ordering of the high-aspect ratio of MoS_2_ layers. This suggests an increase in the specific surface area of the nanocomposite due to the incorporation of MoS_2_ nanoflower into the polymer matrix. The enhanced surface area may provide more active surface sites for electrochemical reactions to occur.

### Optical properties

**Figure 4:**
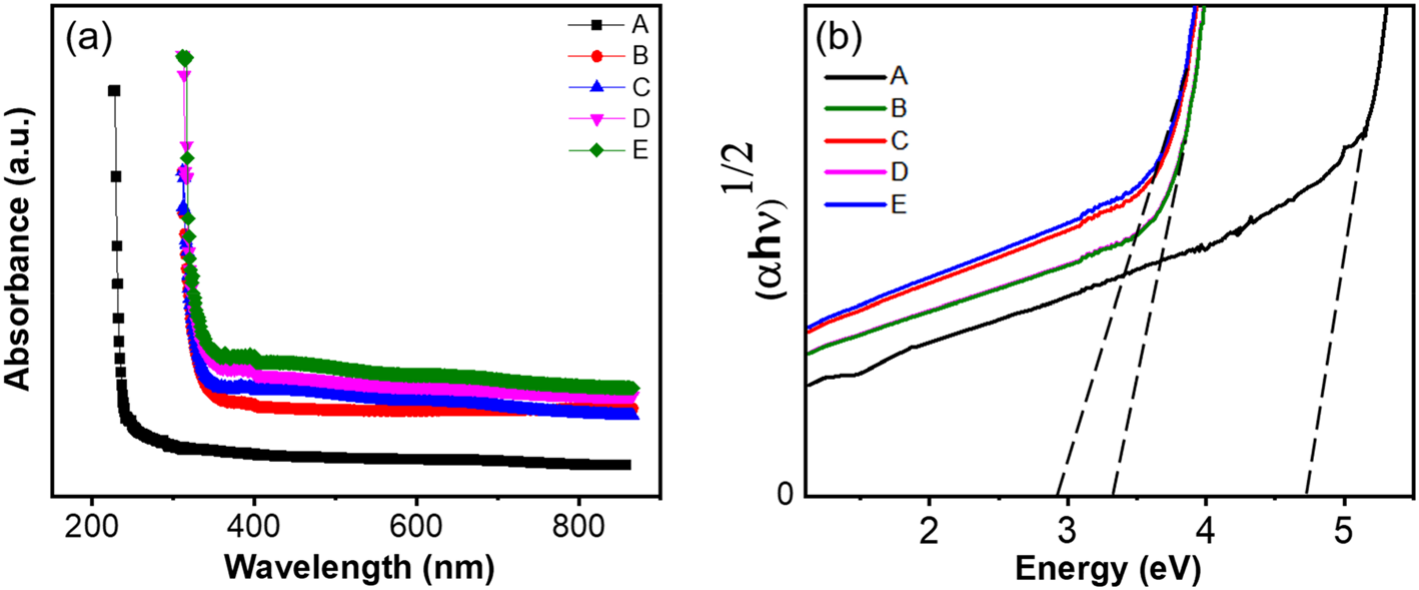
(**a**) Tauc plot for band gap calculation (**b**) band gap variation with the varying concentration of MoS_2_ of PS/rGO/MoS_2_ nanocomposite.

Figure 4a illustrates how the optical absorption of the nanocomposites is influenced by rGO and MoS_2_. The presence of rGO and MoS_2_ in the nanocomposite led to an enhancement in optical absorbance; notably, as the concentration of MoS_2_ increased, the UV light absorption of the nanocomposites also exhibited an increment. The improved UV shielding performance can be ascribed to two contributing factors. Firstly, the even distribution of MoS_2_ nanoflower enables efficient UV light absorption, leading to its conversion into heat. Secondly, the UV light may suffer scattering between the PS/rGO/MoS_2_ interfaces. Through the utilization of absorbance (A) data, the absorption coefficient (α) is computed using the Beer-Lambert equation:

6 $$\alpha = \frac{2.303 A}{d}$$

where *d* is the thickness of the nanocomposites. Figure 4b illustrates how the absorbance changes with wavelength for the PS/rGO/MoS_2_ nanocomposite. The band gap of the nanocomposite can be ascertained from the absorption coefficient following Tauc’s equation^45^:

$$\alpha h\nu = \beta \left( {h\nu - E_{g} } \right)^{n}$$

where* h* is Planck’s constant, *β* is a specimen structure-dependent constant, $E_{g}$ is the band gap energy, and n is an empirical index. To ascertain the band gap, (*αhν*)^1/*n*^ was plotted as a function of *hν*. The optimal fit for the current optical data corresponds to n = 1/2, as depicted in Fig. 4b. The indirect band gap of the polymer nanocomposites is estimated from the extrapolation of linear portions to the energy axis. The calculated values for the band gaps of the PS/rGO/MoS_2_ nanocomposites at different concentrations of MoS_2_ nanofillers are presented in Table 2. From the Table, a downtrend of bandgap from 4.71 to 2.8 eV is observed with the increasing concentration of MoS_2_. The incorporation of MoS_2_ may give rise to additional energy levels in between the conduction and valence bands, consequently leading to a decrease in the nanocomposite's bandgap.

**Table 2:** The optical band gap variations with the variety of MoS_2_ concentrations.

Sample notation	Nanocomposites	Bandgap (eV)	Urbach Energy (eV)
A	Plasticized starch (PS)	4.71	0.23
B	PS/rGO (1%)	3.10	0.25
C	PS/rGO/MoS_2_ (0.25%)	3.00	0.32
D	PS/rGO/MoS_2_ (0.5%)	2.90	0.39
E	PS/rGO/MoS_2_ (1%)	2.80	0.47

**Figure 5:**
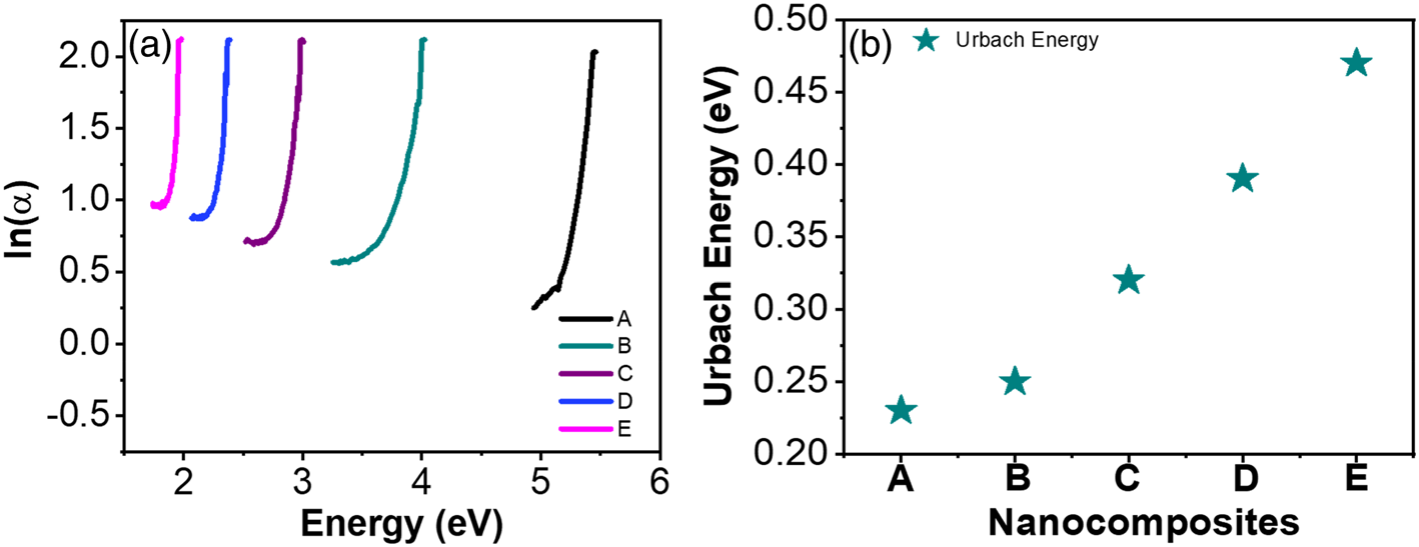
(**a**) Plot of ln(α) versus energy to evaluate the Urbach energy of PS/rGO/MoS_2_ nanocomposites (**b**) variation of Urbach energy as a function MoS_2_ concentration.

Urbach energy of the nanocomposites was studied to further explore the effect of MoS_2_ and rGO nanofiller on the optical bandgap of the starch. During optical transition, the charge carrier may encounter disorders that comes from defect centers and thermal vibration. These disorders create the density of states tailing in between the forbidden energy gap. The defect states tailing is associated with energy which is called Urbach energy, *E*_*U*_. *E*_*U*_ is related to the absorption coefficient by^46^.

7 $$\alpha = \alpha_{0} exp\frac{{hv - E_{g} }}{{E_{U} }}$$

where $\alpha_{0}$ is a constant, *hν* is the photon energy, $E_{g}$ is the bandgap. *E*_*U*_ can be determined from the *log*(*α*(*ν*)) versus *hν* graph. The inverse of the slopes of the straight-line portion of the graphs gives the *E*_*U.*_ The value of *E*_*U*_ obtained for different samples is provided in Table 2 and plotted in Fig. 5. Figure 5 shows that the incorporation of rGO/MoS_2_ nanofillers causes a gradual increase of *E*_*U*_ value, from 0.23 to 0.47 eV. This increase in *E*_*U*_ indicates an increase in defect states into the composite nanomaterials due to the incorporation of nanofillers.

The rise in *E*_*U*_ resulting due to the integration of rGO can be ascribed to the formation of ionic complexes and the disruption and distortions within the host PS polymer. These effects come from the interactions between diverse surface oxygenated functional groups on rGO and PS. This increment also suggests the existence of localized states that may act as trap centers for charge carriers and facilitate the optical transition^47^. The trend of increment of *E*_*U*_ also continues with the loading of MoS_2_ concentrations. This can be attributed to the sulfur vacancy pairs of MoS_2,_ which may introduce additional shallow trap states into the host PS matrix. Furthermore, the Mo and S act as interstitial impurities which occupy the interstitial spaces between the PS matrix. The defects originated by the nanofillers introduce additional energy levels between the forbidden gaps, reducing the optical bandgap.

### Surface wettability

**Figure 6:**
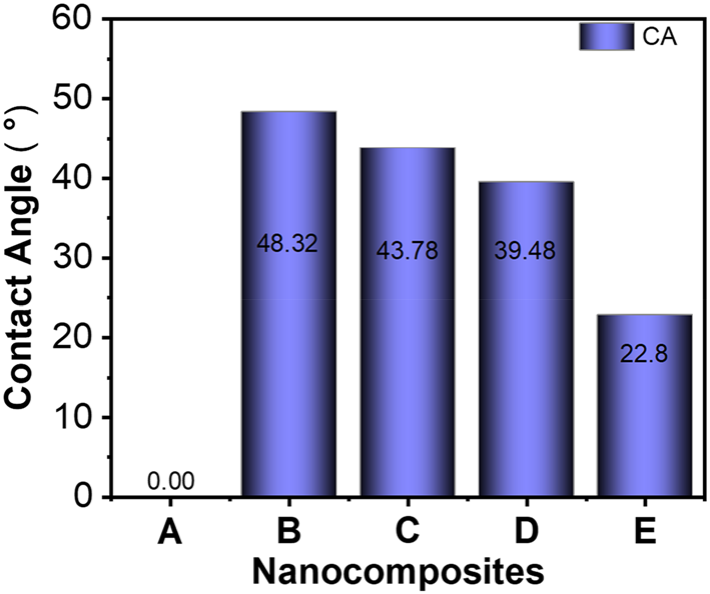
Contact angle (CA) of as-prepared PS/rGO/MoS_2_ nanocomposites, where MoS_2_ concentration were varied.

The contact angle (CA) is used to measure the surface wettability of a material is a critical factor in determining the accessibility of aqueous electrolyte into the electrode. The CA of different samples is presented in Fig. 6. According to Fig. 6, PS shows a 0 °CA, suggesting high surface wettability and surface energy of PS. When it falls onto the PS film, the water gets absorbed immediately due to the roughness. The hydrophilic polar radicals of the constituent molecules of PS immediately absorbed the water that came into the contact. The incorporation of rGO increased the CA of the nanocomposite from 0° to 48.32°. The rGO nanofiller is enriched with non-polar C–C and C=C bonds. These non-polar bonds reduce the affinity of the material for water molecules, leading to the increase of CA^48^. Then, due to the incorporation of MoS_2_ nanofiller,, the value of CA was found to reduce gradually from 48.32° to 22.80°. The observed decrease in CA can be attributed to the presence of a negative charge on the surface of MoS_2_, which comes from their sulfur vacancies and other defects^49^. This negative charge attracts and interacts with the positively charged hydrogen atoms in water molecules, facilitating the adsorption of water and enhancing hydrophilicity. Also, MoS_2_ offers a high surface roughness and is composed of stacked layers that are held together by weak van der Waals forces at the nanoscale^20^. The layers and roughness accommodate water molecules between them, and the water molecules adhere to the surface. So, together with this dispersive interaction of the non-polar functional group, which is driven by the Van der Waals force, increases the surface wettability, leading to hydrophilic behavior.

### Electrochemical properties

The cyclic voltammetry (CV) measurements were done at different scan rates (from 5 to 200 mV s^−1^) to identify the charge storage and charge transfer behaviors of nanocomposite. The CV curve describes the Nernst equation which relates the potential of an electrochemical cell (E) to the standard potential (E°) and the equation is^50^:

8 $$E = E^{o} + \frac{RT}{{nF}} {\text{ln}}\left( {\frac{Ox}{{Red}}} \right)$$

**Figure 7:**
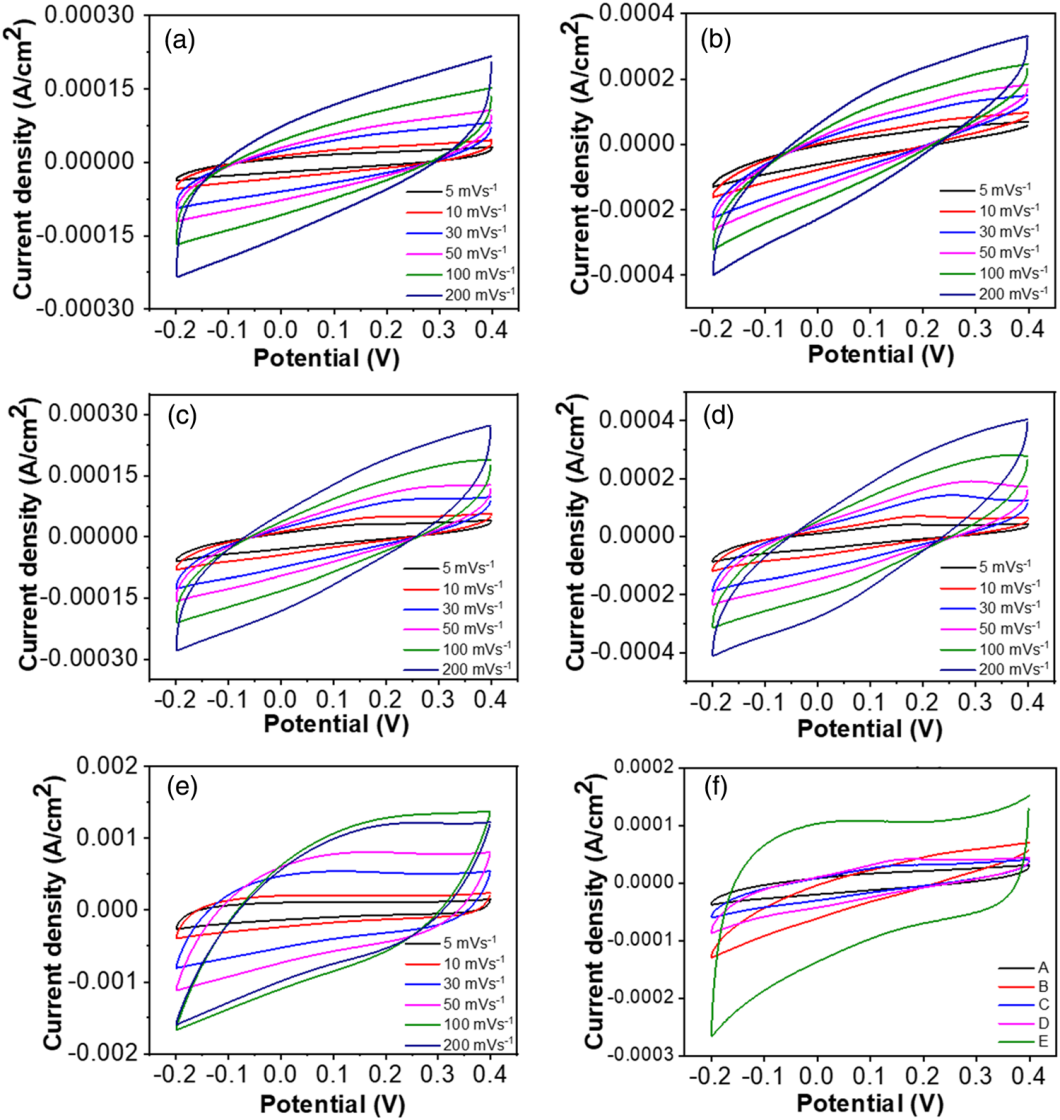
Cyclic voltammetry curve obtained using three electrode system in 0.1 M aqueous solution of KCl within the potential window of 0.2–0.4 V of (**a**) PS (**b**) PS/rGO (1%) (**c**) PS/rGO/MoS_2_ (0.25%) (**d**) PS/rGO/MoS_2_ (0.5%) and (**e**) PS/rGO/MoS_2_ (1%) at different scan rate (**f**) CV data at 5 mV s^−1^.

Figure 7a–e shows the CV curves for PS, PS/rGO, and PS/rGO/MoS_2_ nanocomposites. All of the curves show the sharp increment of current density in the forward scan rate and drop sharply in the reverse scan. This behavior suggests the composites are electrochemically stable as electrode material. The CV curves were found to be changed from a leaf-like shape to a rectangular shape with the addition of rGO and then MoS_2_ into PS. Additionally, a hump of redox peak is observed at 0.138 V with the 0.25% concentration of MoS_2_ (Fig. 7c). This presence of this redox peak suggests the existence of pseudo capacitance^51^. This redox peak was absent in PS and PS/rGO. The rise in the redox peak comes from the active site provided by MoS_2_ for redox reaction via vacancy and defect states. Then for the 0.50% concentration of MoS_2_, the hump is slightly shifted and becomes wide, indicating an increase in the redox reaction rate. But, after the addition of 1% MoS_2_, the curve becomes more rectangular, which is caused by EDLC behavior. This distortion confirms the applicability of a pseudo capacitor and EDLC^52^.

Figure 7f shows the CV curves for all the nanocomposites at a fixed scan rate of 5 mV s^−1^. The quasi-rectangle CV curves of the PS/rGO/MoS_2_ nanocomposite are greater than that of pure PS and PS/rGO, which indicates that the MoS_2_ boosts capacitive performance compared to pure PS. The intercalating complex reaction through the layer structure enhanced the spacing of MoS_2_, which decreased the energy barrier of ion diffusion, increased the ion intercalation, and boosted the capability of charge storage^53^.

**Figure 8:**
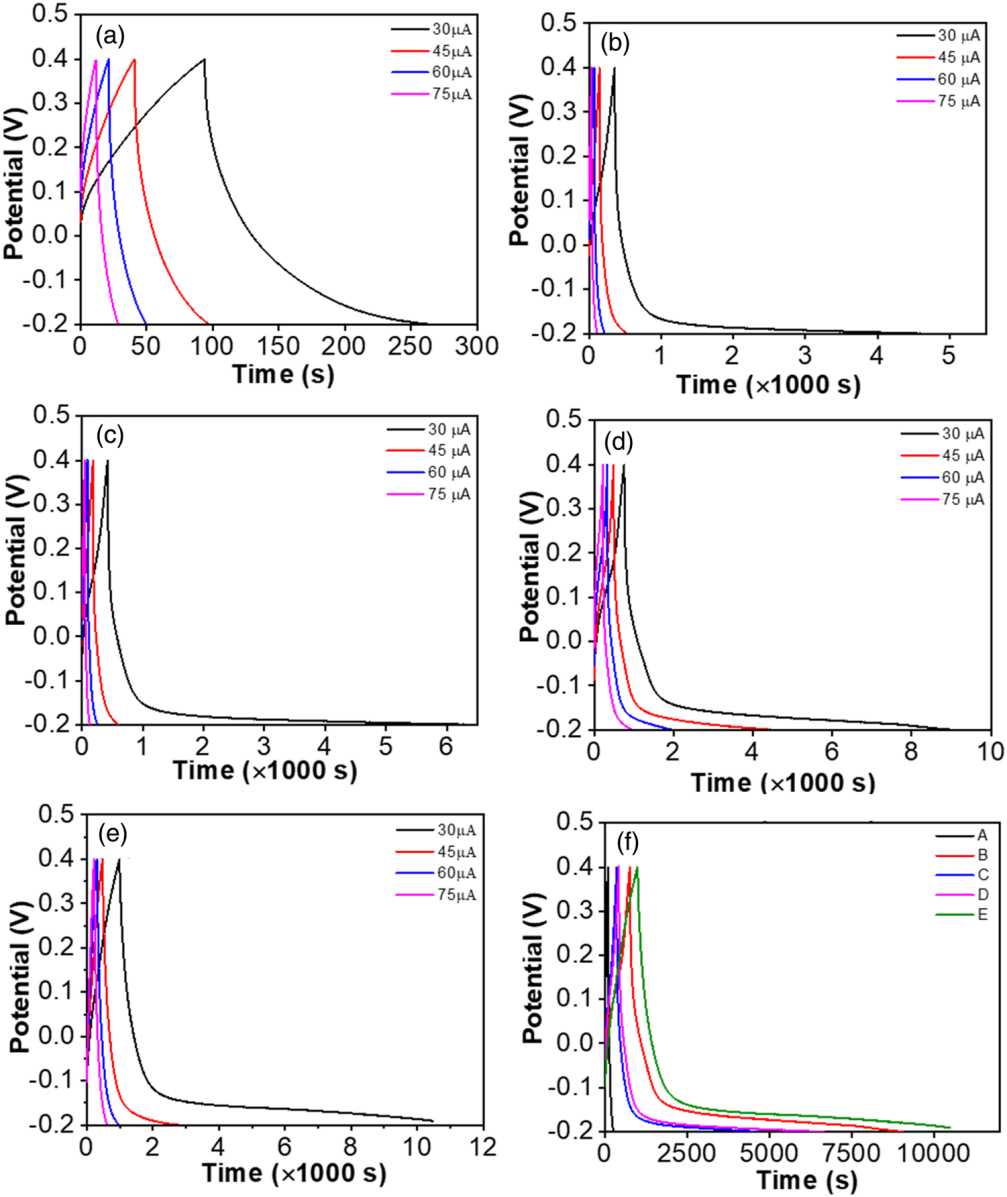
Galvanostatic charging discharging curve of (**a**) PS (**b**) PS/rGO (1%) (**c**) PS/rGO/MoS_2_ (0.25%) (**d**) PS/rGO/MoS_2_ (0.5%) and (**e**) PS/rGO/MoS_2_ (1%) at different current density (**f**) GCD curve at a current density of 0.1 mA g^−1^.

Figure 8 demonstrates the galvanostatic charging-discharging (GCD) curves at various current densities in the range of 0.1–3 mA g^−1^. The discharge time is increasing with the decrease of current density for all five nanocomposites. This is because, at lower current density, a smaller number of ions can come close to a certain portion of surface area and can diffuse easily within electrolytes^54^. On the other hand, at high current density, ion traffic occurs at the same surface; thus, the charging-discharging rate accelerates, and a short discharge time is observed. In addition, with the lower current density, the electrolyte can take place at the empty sites of the electrode; hence the smooth ion-electrolyte intercalation also takes part in the increment of discharge time and eventually higher capacitive behavior^55^. It was observed from Fig. 8f that the discharge time extends as the MoS_2_ content increases. Additionally, all the GCD curves deviate from the symmetric triangular shape, suggesting the presence of a pseudocapacitive charge storage mechanism.

From Table 3 and Fig. 8, a comparison of energy storage performance has been estimated. Specific capacitance was calculated by the following equation:

9 $$C_{s} = \frac{I\Delta t}{{m\Delta V}}$$

**Table 3:** Specific capacitance, energy density and power density of PS and PS/rGO/MoS_2_ nanocomposites at a current density of 0.10 mA cm^−2^.

Sample	Specific capacitance (F g^−1^)	Energy density (Wh kg^−1^)	Power density (W kg^−1^)
PS	2.78	0.50	11.12
PS/rGO (1%)	35.67	6.42	2.79
PS/rGO/MoS_2_ (0.25%)	76.35	13.74	11.55
PS/rGO/MoS_2_ (0.5%)	86.42	15.56	9.01
PS/rGO/MoS_2_ (1%)	124.91	22.48	8.52

where $C_{s}$ = Specific capacitance, *I* = Current, *Δt* = Discharge time, *m* = Deposited mass, Δ*V* = Potential window.

Energy density (*E*) and power density (*P*) were also calculated by calculation of specific capacitance using the following relation:

10 $$E = \frac{{C_{s} \left( {\Delta V} \right)^{2} }}{2}$$

11 $$P = \frac{E}{t}$$

These values for each nanocomposite have been presented in Table 3. The specific capacitance of PS was estimated to be 2.78 F g^−1^. The granule microstructure highly resists ion transportation. The various range of granules, together with the grain boundary of PS, hinders the charge transportation process^56^. In the plasticizing process, the disruption of the amylose and amylopectin chain occurs due to the high temperature. This retrogradation may have an impact on the specific capacitance of starch. Then, with the addition of rGO, the specific capacitance increases from 2.78 to 35.67 F g^−1^. The layer structure and its wide surface facilitate charge transportation. This accessible area between the electrolyte and active material improves the capacitive performance. Additionally, the layered structure of rGO reduces the diffusion length of electrolyte ions, further boosting capacitance^57^.

The incorporation of MoS_2_ nanofiller was found to improve the specific capacitance of the nanocomposite. Also, the specific capacitance was found to increase with the concentration of MoS_2_ loading into the PS matrix. The incorporation of 1% MoS_2_ nanoflower was found to provide specific capacitance as high as 124 F g^−1^, which is 62 times higher than that of the PS. The specific capacitance obtained for the ternary PS/rGO/MoS_2_ nanocomposite is also larger than that of the binary PS/rGO nanocomposites estimated by Mollick et al.^17^. Such an improvement of the specific capacitance of the PS matrix due to the incorporation of MoS_2_ can be attributed to a number of factors: Firstly, the combined higher specific surface area of rGO and MoS_2_ together expands the functional area of the interface between the nanocomposite and electrolyte^58^. Also, the porous structure of MoS_2_ nanoflower can enhance the specific capacitance. Micropores and mesopores can enlarge the electroactive region and accelerate ion diffusion^59^. Furthermore, an increase in MoS_2_ concentration creates more active sites, which give the free pathway of ions, hence, enhancing the electrode reaction kinetics^60^. Additionally, MoS_2_ concentration also offers improved surface wettability which enhances the electrical conductivity.

Additional insights were gathered from Electrical Impedance Spectroscopy (EIS) to further analysis of the ion migration and charge transfer process. The impedance (Z) is related to alternating voltage as such:

12 $$Z \left( \omega \right) = \frac{v \left( \omega \right)}{{I \left( \omega \right)}}$$

where ω = angular frequency = 2πf, f = frequency.

The alternating potential and the corresponding current create sinusoidal output and can be expressed as:

13 $$\Delta V \left( \omega \right) = \Delta V_{max} e^{j\omega t}$$

14 $$\Delta I \left( \omega \right) = \Delta I_{max} e^{{j\left( {\omega t + \varphi } \right)}}$$

Here, φ = Phase difference between the current and voltage.

Then the impedance become:

15 $$Z = Z \left( \omega \right)e^{ - j\varphi }$$

Using Euler theorem this expression become:

16 $$Z \left( \omega \right) = Z^{\prime } \left( \omega \right) + jZ^{\prime \prime } \left( \omega \right)$$

where $Z^{\prime } \left( \omega \right)$ = Real impedance, $Z^{\prime \prime } \left( \omega \right)$ = Imaginary impedance.

**Figure 9:**
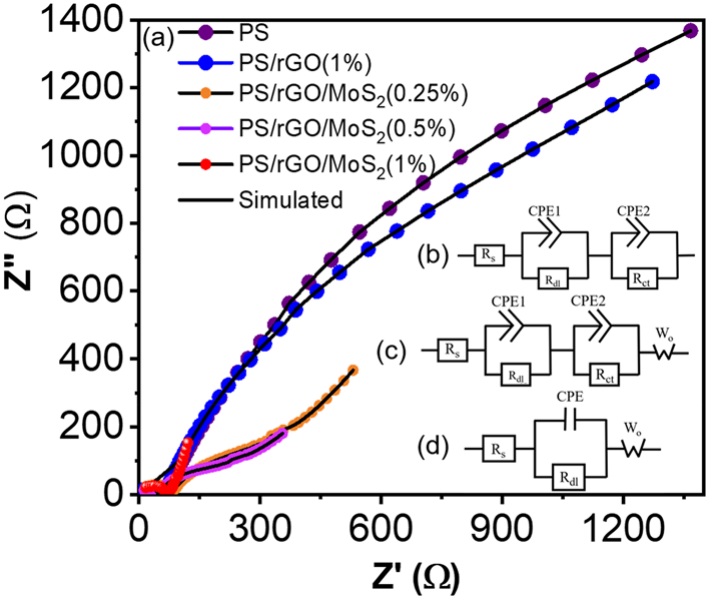
(**a**) The Nyquist plot with the best fitted simulation curve (**b**) circuit of PS, PS/rGO, (**c**) circuit of PS/rGO/MoS_2_ (0.25 and 0.5%), (**d**) circuit of PS/rGO/MoS_2_ (1%) nanocomposites performed at 0.01 Hz to 10^6^ Hz.

Therefore, Fig. 9a is representing the EIS Nyquist plot where real impedance *(Z′*) is plotted against corresponding imaginary impedance (*Z″*). The dotted line represents the experimental data, whereas the simulation data is presented as a solid line. The equivalent circuit for each nanocomposite was simulated from the plot and presented in the inset of Fig. 9. Zview software was used for the simulation and circuit fitting. Corresponding circuit parameters of the equivalent circuit are presented in Table 4.

**Table 4:** Electrochemical impedance variations within the nanocomposites.

Sample	Solution resistance, R_s_ (Ω)	Double layer resistance, R_dl_ (Ω)	Charge transfer resistance, R_ct_ (Ω)
PS	11	40	4574
PS/rGO (1%)	10	50	1958
PS/rGO/MoS_2_ (0.25%)	06	67	497
PS/rGO/MoS_2_ (0.5%)	09	37	293
PS/rGO/MoS_2_ (1%)	10	55	–

Figure 9b presents the fitted circuit for PS and PS/rGO. The equivalent circuit consists of a series resistance, *R*_*s*_ is connected to a constant phase element, CPE which is parallelly connected to a double layer resistance, *R*_*dl,*_ and charge transfer resistance, *R*_*ct*_*.* CPE arises due to the nonideal capacitive behavior of the electrochemical system. The R_dl_ comes from the grain contribution, and R_ct_ comes from the grain boundary contribution. High *R*_*ct*_ is observed for PS compared to PS/rGO which is 4574 Ω and 1958 Ω, respectively. This high resistance of PS comes from the rough and granule structure which creates boundaries that interrupt the free movement of ion transportation^55,61^. Additionally, PS consists of chained amylose and amylopectin, which reduce the area of ion movement^62^. On the contrary, the layer structure and the wide surface area of rGO reduce the boundary and facilitate ion transportation^63^. Then Fig. 9c represents the equivalent circuit for the PS/rGO/MoS_2_ (0.25%, and 0.5%). The fitted circuit changed with a fourth component, the Warburg coefficient (*W*_*o*_). The Warburg coefficient (*W*_*o*_) represents the degree of diffusion resistance within the inhibitor PS film. The gradual decrease of *R*_*ct*_ is also observed from 497 to 293 Ω with the loading of 0.25%, and 0.50% of MoS_2_ concentration respectively. Figure 9d represents the equivalent circuit for PS/rGO/MoS_2_ (1%). The deletion of the third component is observed, which indicates a zero *R*_*ct*_ value. This suggests a gradual decrease of the grain resistance due to the incorporation of MoS_2_ nanofiller for charge transferring within the electrolyte or inter-intra layer^64^. This decrement of resistance is attributed to the fact that with the addition of MoS_2,_ the functioning area is gradually increased, thus facilitating the charge transportation and decrease, eventually diminishing the contact resistance^65^. The improved wettability also facilitates the intercalation with electrolytes^51^. The reduction of *W*_*o*_ is also found compared to PS/rGO/MoS_2_ (0.25% and 0.5% films). The lower the value, the less difficult it is for ions to diffuse through the pores in the inhibitor PS films. This suggests that the PS films are less porous or can have pores with smaller equivalent diameters. It also suggests the material is less corrosive^66^.

**Figure 10:**
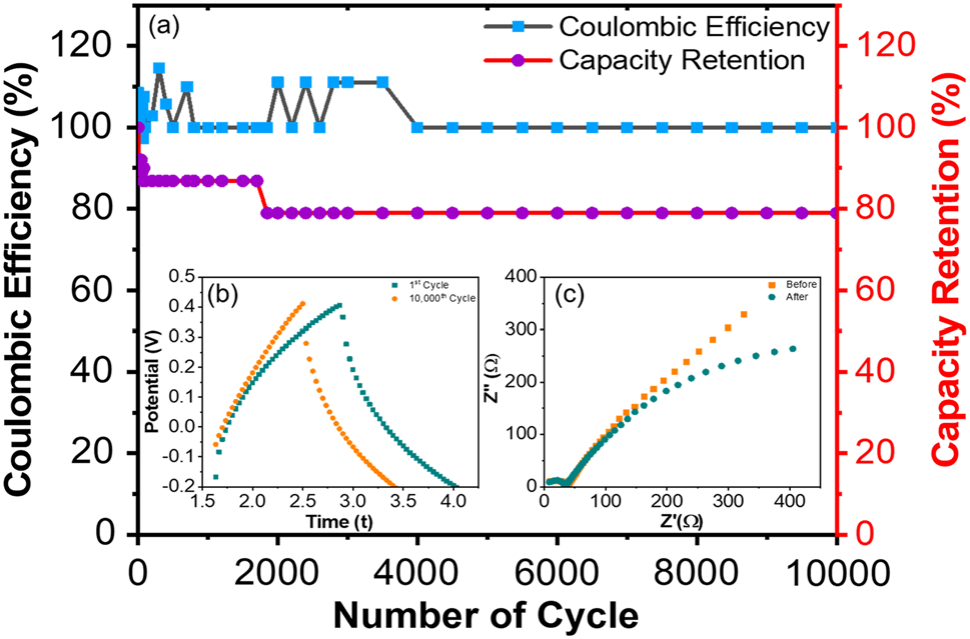
(**a**) The coulombic efficiency and capacitive retention for 10,000 cycles from GCD (**b**) discharging time for 1st and 10,000th cycle and (**c**) electrochemical impedance spectra (EIS) curve for the 1st and 10,000th cycle of GCD curve.

Capacitive retention is the measure of the ability of a device to retain stored energy during an open circuit test. Figure 10a depicts the cyclic stability: Coulombic efficiency and capacity retention of PS/rGO/MoS_2_ nanocomposites as an electrode material. Coulombic efficiency (*η*) is calculated by the following equation:

17 $$\eta \left( \% \right) = \frac{Discharge time}{{Charge time}} \times 100$$

Then capacitive retention was calculated:

18 $$Retention \left( \% \right) = \frac{Specific\;capacitnce\;of\;nth \;cycle}{{Specific\;capacitance\;of\;1st\; cycle}} \times 100$$

It is observed from the Fig. 10a that 100% efficiency was achieved after completing 10,000 cycles. However, cyclic retention gets reduced to 86% after 100 cycles, and then after 2000 cycles, it reduces to ~ 80%. However, this 80% was consistent till 10,000 cycles. This first reduction to 86% can be attributed to the fact of the saturation of active site on the PS/rGO/MoS_2_ film surface during the charge–discharge cycle. Furthermore, the slight decrease to 80% is due to the structural breakdown and delamination of the film surface in the aqueous solution of KCl, and the ion may have been trapped in the layer of rGO/MoS_2_ due to the repetition of charging and discharging cycles^67^. It has been observed that a 100% Coulombic efficiency was achieved even after completing 10,000 cycles.

Figure 10b represents the EIS curve of the 1st and 10,000th cycle. After undergoing 10,000 cycles of the electrode material, the radius of the impedance spectrum's semi-circle was found to be diminished compared to that observed during the first cycle. This reduction of radius suggests the reduction of internal resistance. The findings indicate that up to 10,000 cycles of operation, this electrode displays an increased electrical conductivity and demonstrates rapid electron-conducting capabilities at the interface. This reduction of internal resistance can be ascribed to the electrochemical stimulation of the active material, decreased aggregation of the active material throughout cycling, and an enhanced interface^68^. From Fig. 10c, it is observed that the discharge time drains faster after uninterrupted 10,000 cycles.

From this study, it is observed that the incorporation of MoS_2_ nanoflower produces defect sites into the nanostructure that offer more active sites. Furthermore, the enhancement of the surface area due to the MoS nanofiller helps facilitate the increased number of active sites and improves the electrochemical performance of the nanocomposites. Thus forming a ternary nanocomposite by incorporating MoS_2_ nanofillers into the PS/rGO composites will offer enhanced capacitive performance by keeping the concentration of rGO low. The improved electrochemical performance of nanocomposite can therefore be attributed to the synergistic effect of the layered structured MoS_2_ and rGO nanofillers, which improve the crystallinity, incorporate defect sites, reduce the contact resistance, and provide a large surface area.

## Conclusions

In conclusion, biodegradable PS/rGO/MoS_2_ nanocomposites have been prepared following a facile solution casting method. The structural and surface study demonstrates improved interaction between PS and rGO/MoS_2,_ which leads to the reduction of composite crystallinity and reveals the presence of defects. From the UV–vis study, the band gap reduction was found together with the increment of defects, which was confirmed by the Urbach energy analysis that comes from the contribution of MoS_2_ loading. Contact angle study shows the improvement of surface wettability due to the incorporation of MoS_2_. The PS/rGO/MoS_2_ nanocomposites provide specific capacitance as high as 124 F g^−1^, about 60 times higher than that of PS. The nanocomposite also provides excellent cyclic stability of about ~ 85% retention of the capacitance up to 10,000 cycles of operation. The notable decrease in bandgap and crystallinity leads to the adsorption of electrons and ions into the biopolymer matrix, resulting in enhanced electrochemical performance. Furthermore, the structural and sulfur drive defect that comes from the stacked MoS_2_/rGO nanosheets leading to very high surface area, enhanced electrochemical conductivity, and interaction with the –OH functional group provided by the nanofiller improved the electrochemical performance of the nanocomposite. This research has the potential to provide a straightforward and cost-effective method for creating environmentally friendly energy storage devices, which are essential for ensuring a sustainable energy future.

### Supplementary Information

Supplementary Information.

## Data Availability

All data generated or analyzed during this study are included in this published article.
