# Supplementary material for: Improved electrochemical performance of bio-derived plasticized starch/ reduced graphene oxide/ molybdenum disulfide ternary nanocomposite for flexible energy storage applications
Source: Sci Rep. 2023 Nov 28;13:20967. doi: 10.1038/s41598-023-48326-8 (PMC10684543; doi:10.1038/s41598-023-48326-8)
Supplement: Supplementary file 1 — Supplementary Information. [file 41598_2023_48326_MOESM1_ESM.docx]

**Improved electrochemical performance of bio-derived plasticized starch/** **reduced graphene oxide/** **molybdenum disulfide ternary nanocomposite for flexible energy storage applications**

Eashika Mahmud, Muhammad Rakibul Islam^*^

*Department of Physics, Bangladesh University of Engineering and Technology (BUET), Dhaka, Bangladesh,*

**Corresponding Author:* [*rakibul@phy.buet.ac.bd*](mailto:rakibul@phy.buet.ac.bd)

**Extraction of starch**

Fresh potatoes were first collected from the local market. Then cleaned, peeled, and grated at the laboratory, maintaining all the cautions. The grated potato was put into a beaker, and Deionized (DI) water was poured into the beaker. DI water was added to remove the cell debris. Then, the solution of potato water was stirred with a glass rod. This stirring separated the starch particle, which fell down in the beaker. Before the liquid color became brown, the crumpled potato was separated from the liquid and steadfast in the beaker. After keeping the beaker steady for around 15 minutes at room temperature, the white milky starch settled as sediment in the beaker. Then, the sediment starch was collected by removing the water. The wet starch cake was separated from the beaker and kept in a Petri dish. This wet product was washed with DI water 2/3 times to make sure of purity. Finally, the pure starch cake was dried in an oven at 60 ℃ for several hours and then grinded with a mortar pestle to get the fine starch powder.


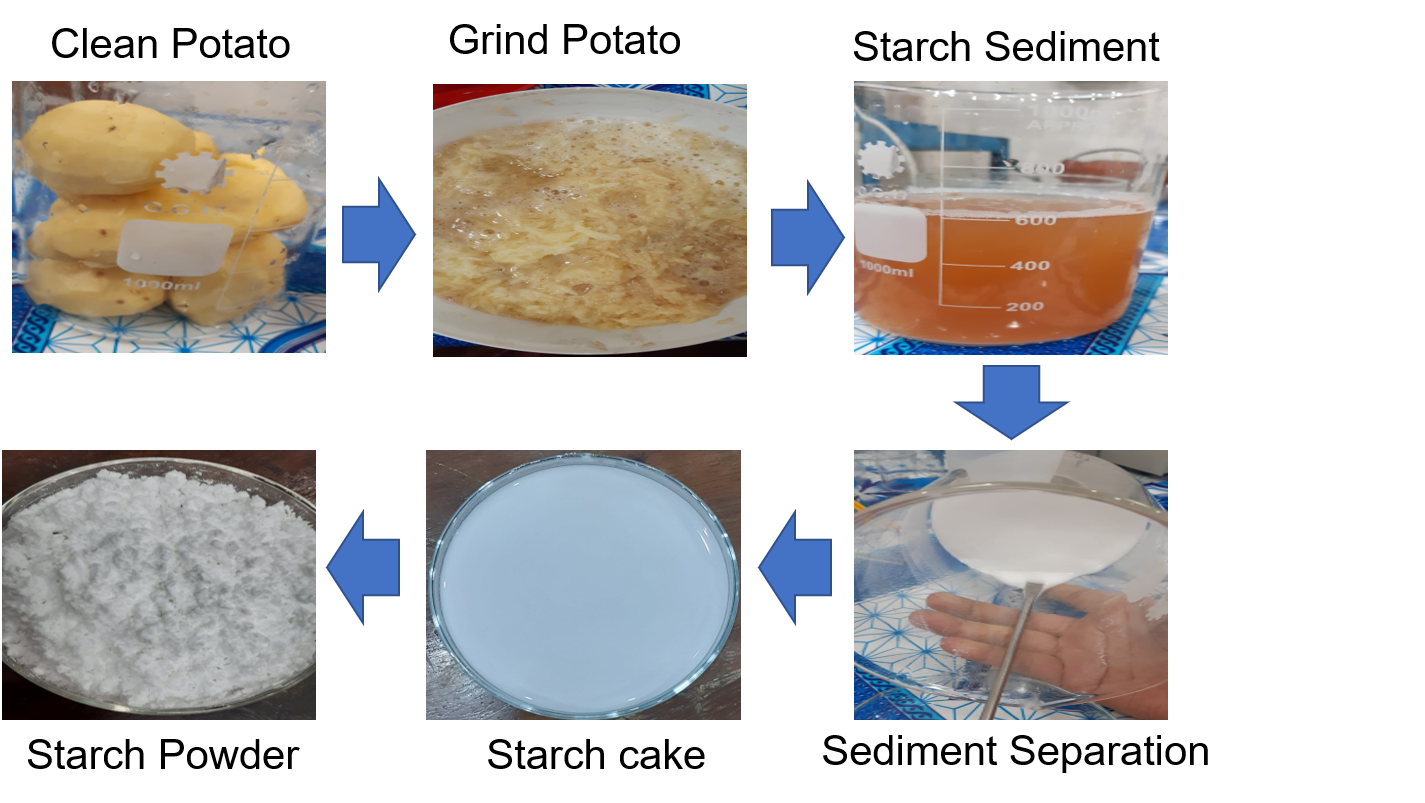


**Fig S1.** Flow chart showing the different steps involved in the synthesis of starch


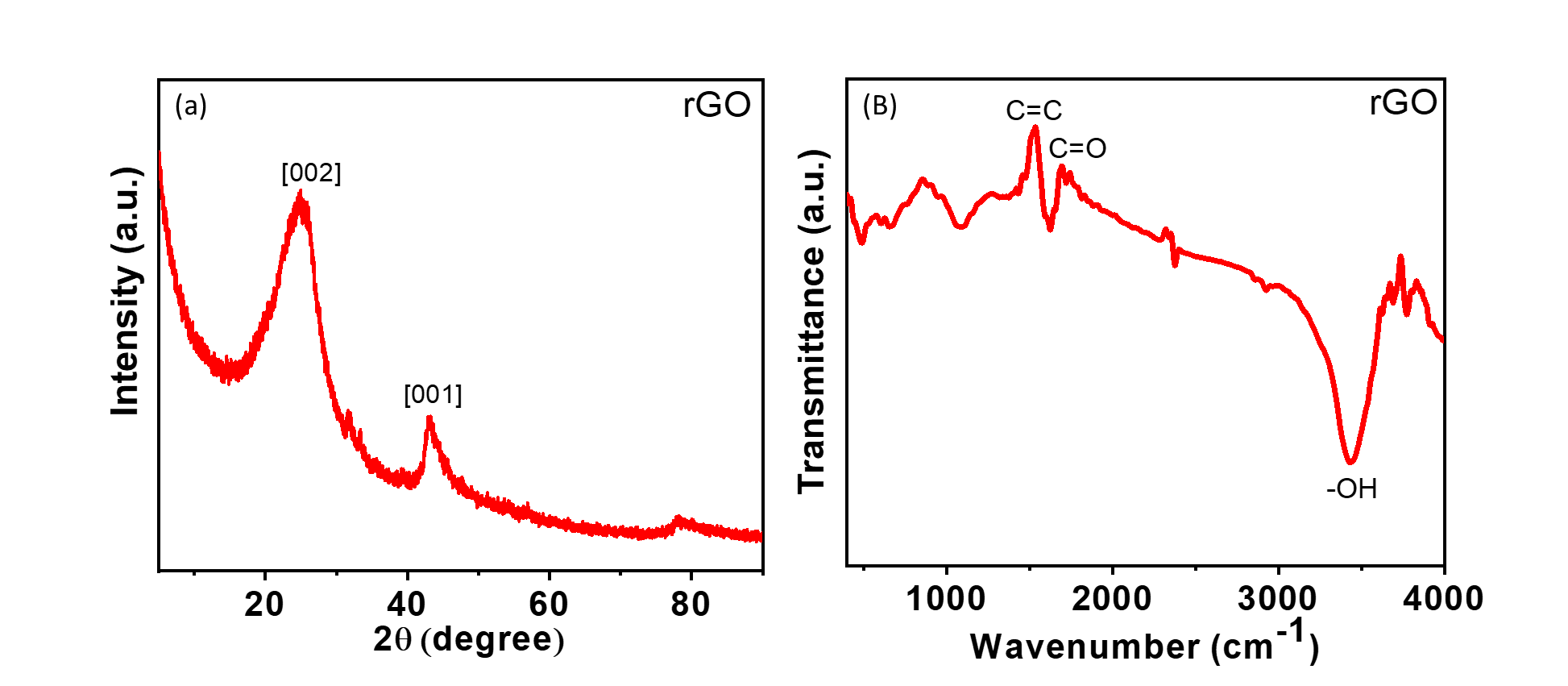


**Fig S2.** (a) XRD and (b) FTIR spectrum of rGO


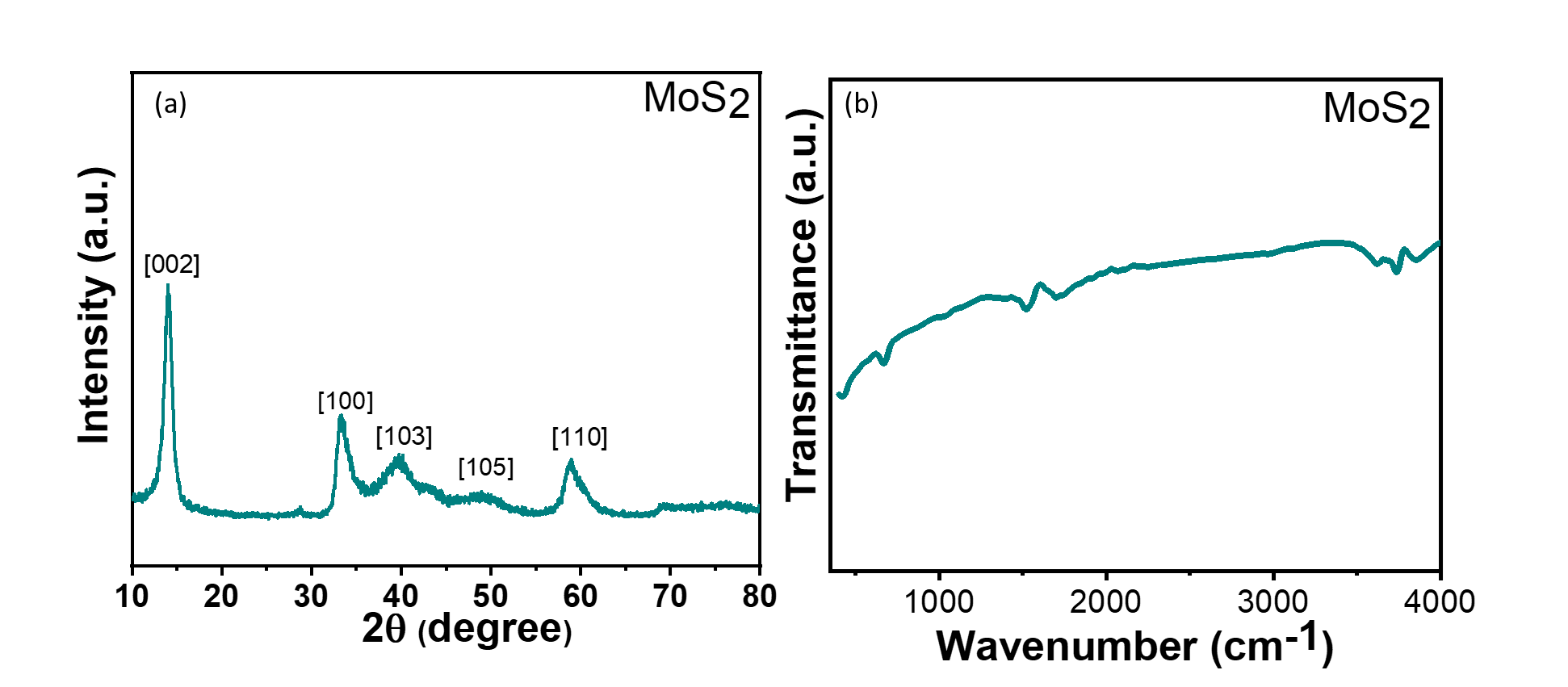


**Fig S3.** (a) XRD and (b) FTIR spectrum of rGO
